# P-804. Shorter vs. Longer Duration of Antibiotics in Pediatric Uncomplicated Gram Negative Bacteremia

**DOI:** 10.1093/ofid/ofae631.996

**Published:** 2025-01-29

**Authors:** Alec Wesolowski, Marisol Fernandez, Kavitha Gilroy

**Affiliations:** Dell Children's Medical Center, Austin, Texas; Dell Children's Medical Center of Central Texas, Dell Medical School at UT Austin, Austin, Texas; Dell Children's Medical Center, Austin, Texas

## Abstract

**Background:**

Guidelines for uncomplicated gram negative bacteremia recommend 7-14 days of antibiotics; however there is limited pediatric evidence on duration of therapy. Studies in adult patients support a shorter duration of treatment and earlier transition to oral antibiotics. The primary objective of this study was to describe the local practice of a free standing children’s hospital in treating uncomplicated gram negative bacteremia in pediatric patients.

**Methods:**

This single-center, retrospective study included pediatric patients from January 2022 to December 2023 with uncomplicated gram negative bacteremia. Those with chronic medical conditions, immunocompromised status, and polymicrobial bacteremia with mixed gram positive and gram negative organisms were excluded. Short-course antibiotic duration was defined as 7-10 days and long-course antibiotic duration was defined as > 10 days. Outcomes assessed included recurrent infection, readmission, length of stay, and adverse events between the two groups.

**Results:**

156 positive blood cultures were identified and 31 patients met inclusion criteria. 16 patients had a total antibiotic duration of 7-10 days (mean 7.8 days) compared to 15 patients with an antibiotic duration >10 days (mean 14.6 days). The average length of hospitalization was 6.8 days in the 7-10 day group and 14.2 days in the >10 day group. The primary organism identified was *E. coli* (64.5%) from a urinary source (77.4%) with a majority of patients transitioning to oral antibiotics (61.3%) to complete therapy. Neither group developed a recurrent infection within 30 days; however, 3 patients in the >10 day group had a readmission within 30 days. More adverse events occurred in patients that received a longer duration of therapy (20%) vs those that received a shorter duration of therapy (6.3%).

**Conclusion:**

7-10 days of total antibiotics may be appropriate in immunocompetent pediatric patients with uncomplicated gram negative bacteremia. Shorter duration of antimicrobial therapy is associated with decreased length of hospitalization and fewer adverse reactions without increased risk of recurrent infection.

**Disclosures:**

**All Authors**: No reported disclosures

